# Targeted delivery of GABA via ultrasound-induced blood-brain barrier disruption blocks somatosensory-evoked potentials

**DOI:** 10.1186/2050-5736-3-S1-P28

**Published:** 2015-06-30

**Authors:** Nathan McDannold, Yong-Zhi Zhang, Chanikarn Power, Costas Arvanitis, Natalia Vykhodtseva, Margaret Livingstone

**Affiliations:** 1 Brigham & Women’s Hospital/Harvard Medical School, Boston, Massachusetts, United States; 2 Harvard Medical School, Boston, Massachusetts, United States

## Background/introduction

This study investigated whether targeted delivery of the inhibitory neurotransmitter gamma-Aminobutyric acid (GABA), a small molecule (103 Da) that normally does not reach the brain with systemic administration, can temporarily block brain activity after ultrasound-induced blood-brain barrier (BBB) disruption.

## Methods

Focused ultrasound exposures (10 ms bursts applied at 1 Hz for 60 s; pressure amplitude in water: 0.64-0.71 MPa) were delivered immediately after the administration of Definity microbubbles (20 or 40 μl/kg) to disrupt the BBB. The focal point was targeted to 10 overlapping targets on and around the somatosensory motor cortex (2 mm lateral, 2 mm posterior to bregma) under MRI guidance in 5 rats. BBB disruption was confirmed using Gd-DTPA MRI contrast agent (Magnevist, 0.25 ml/kg). After the sonications, electrodes were implanted into the thigh muscle to electrically stimulate the sciatic nerve (stimulation voltage: 9-20 V). Somatosensory evoked potentials (SSEP) were recorded transcranially before and after intravenous GABA at doses ranging from 0.8 to 519 mg/kg. The SSEP recordings were repeated in two animals at 24h after sonication and one animal at 5 days. SSEP measurements were obtained in two control animals who received GABA but not ultrasound-induced BBB disruption.

## Results and conclusions

The amplitude of the SSEP recordings in the animals who received GABA and ultrasound-induced BBB disruption were reduced by 6-94%. There was a good correlation (R²: 0.79) between the percent suppression in SSEP magnitude and GABA dose. This suppression also correlated with the degree of BBB disruption – for similar GABA doses, animals that had less suppression also had weaker enhancement in MRI after Gd-DTPA administration. For relatively small doses of GABA (0.8-64 mg/kg), the duration of the suppression lasted between 2-20 min, with higher doses resulting in longer durations. At high doses (422 mg/kg and above) the suppression lasted the length of the recordings (up to 100 min). No suppression was observed after GABA administration in control animals or in recordings performed 24h or 5d after the sonications. This work demonstrates an alternative ultrasound-based method to block neuronal function. It has some advantages over other ultrasound “neuromodulation” techniques: the ability to use contrast MRI to definitively confirm where the suppression is targeted, the ability to titrate the GABA dose to control the level of suppression, and the ability to recover the animal from anesthesia and transiently block function in a freely behaving animal. Since the effects of GABA and other neurotransmitters have been studied for decades, the mechanisms of the suppression are well understood. We anticipate that the use of GABA and other neurotransmitters in this way can be useful for noninvasively mapping brain function and potentially for surgical planning or novel therapies.

